# The Effect of Anandamide on Uterine Nitric Oxide Synthase Activity Depends on the Presence of the Blastocyst

**DOI:** 10.1371/journal.pone.0018368

**Published:** 2011-04-29

**Authors:** Micaela S. Sordelli, Jimena S. Beltrame, Juliana Burdet, Elsa Zotta, Romina Pardo, Maximiliano Cella, Ana M. Franchi, Maria Laura Ribeiro

**Affiliations:** 1 Laboratorio de Fisiología y Farmacología de la Reproducción, CEFYBO (CONICET – UBA), Buenos Aires, Argentina; 2 Laboratorio de Fisiopatogenia (Departamento de Fisiología, UBA), Buenos Aires, Argentina; 3 Laboratorio de Fisiopatología Molecular (Cátedra de Fisiopatología, Departamento de Cs. Biológicas, Fac. de Farmacia y Bioquímica, UBA), Buenos Aires, Argentina; 4 Laboratorio de Fisiopatología de la Preñez y el Parto, CEFYBO (CONICET – UBA), Buenos Aires, Argentina; Statens Serum Institute, Denmark

## Abstract

Nitric oxide production, catalyzed by nitric oxide synthase (NOS), should be strictly regulated to allow embryo implantation. Thus, our first aim was to study NOS activity during peri-implantation in the rat uterus. Day 6 inter-implantation sites showed lower NOS activity (0.19±0.01 pmoles L-citrulline mg prot^−1^ h^−1^) compared to days 4 (0.34±0.03) and 5 (0.35±0.02) of pregnancy and to day 6 implantation sites (0.33±0.01). This regulation was not observed in pseudopregnancy. Both dormant and active blastocysts maintained NOS activity at similar levels. Anandamide (AEA), an endocannabinoid, binds to cannabinoid receptors type 1 (CB1) and type 2 (CB2), and high concentrations are toxic for implantation and embryo development. Previously, we observed that AEA synthesis presents an inverted pattern compared to NOS activity described here. We adopted a pharmacological approach using AEA, URB-597 (a selective inhibitor of fatty acid amide hydrolase, the enzyme that degrades AEA) and receptor selective antagonists to investigate the effect of AEA on uterine NOS activity *in vitro* in rat models of implantation. While AEA (0.70±0.02 vs 0.40±0.04) and URB-597 (1.08±0.09 vs 0.83±0.06) inhibited NOS activity in the absence of a blastocyst (pseudopregnancy) through CB2 receptors, AEA did not modulate NOS on day 5 pregnant uterus. Once implantation begins, URB-597 decreased NOS activity on day 6 implantation sites via CB1 receptors (0.25±0.04 vs 0.40±0.05). While a CB1 antagonist augmented NOS activity on day 6 inter-implantation sites (0.17±0.02 vs 0.27±0.02), a CB2 antagonist decreased it (0.17±0.02 vs 0.12±0.01). Finally, we described the expression and localization of cannabinoid receptors during implantation. In conclusion, AEA levels close to and at implantation sites seems to modulate NOS activity and thus nitric oxide production, fundamental for implantation, via cannabinoid receptors. This modulation depends on the presence of the blastocyst. These data establish cannabinoid receptors as an interesting target for the treatment of implantation deficiencies.

## Introduction

About half of all conceptuses are lost before the expected term, thus making human reproduction a rather inefficient process [1]. *In vitro* fertilization techniques (IVF) also result in a high frequency of spontaneous abortions [1]. For IVF, the clinical pregnancy rate per aspiration and per transfer is 26.0 and 29.5%, respectively [2]. Implantation seems to be the bottle neck for the improvement of these techniques.

Several reports point to a role for nitric oxide (NO) and anandamide in implantation. NO is produced by conversion of oxygen and L-arginine to NO and L-citrulline. This reaction is catalyzed by nitric oxide synthase (NOS), of which there are three different isoforms: endothelial nitric oxide synthase (eNOS), inducible nitric oxide synthase (iNOS) and neuronal nitric oxide synthase (nNOS) in almost all types of cells [3]. Purcell and colleagues described that at implantation labelled iNOS cells are within the decidua and the ectoplacental cone, while eNOS is localized in vessels of the primary decidual zone [4]. Both iNOS and eNOS expression are higher at implantation sites. Neuronal NOS is localized mainly in the mesometrium and myometrium and does not appear to change throughout peri-implantation. Moreover, *in utero* administration of L-NAME, a non selective NOS inhibitor, or of NO-donors during the pre-implantation phase reduces implantation rates [5], [6]. All together these evidences strongly suggest that optimal levels of NO are crucial for endometrial function and embryo implantation.

Anandamide (N-arachidonoylethanolamine, AEA) is an endogenously produced cannabinoid-like lipid mediator [7] that mimics several actions of the natural *Cannabis sativa* component Δ^9^-tetrahydrocannabinol, which accounts for the majority of the reproductive hazards in marijuana users [8], [9]. AEA binds to and activates two classical G protein (Gi/o)–coupled cell-surface cannabinoid receptors, cannabinoid receptor type 1 (CB1) and type 2 (CB2) [10], [11]. It has been described that high AEA levels are detrimental for implantation [12] and embryo development [13]. However, the fact that the uterus contains the highest concentrations of AEA yet discovered in mammalian tissues [12], [14] suggests that it might play a role in reproduction. The groups of Paria et al. and Guo et al. were the first ones to report that the mouse uterus has the capacity to synthesize AEA [14], [15]. We have recently found that the rat uterus also has an AEA-synthesizing capacity [16], and as previously described in mice [14], [15], AEA production in the rat uterus is spatiotemporally regulated during implantation, being lower in the receptive uterus and at implantation sites.

In other systems different from the uterus, it has been described that while CB1 activates NOS, CB2 inhibits it [17], [18]. Particularly, in human endothelial cells from various blood vessels, CB1 immunoreactive components are present as is its coupling to anandamide-stimulated NOS-derived NO production [19]. The modulation of vascular diameter and vascular tone by NO represents a crucial point of interest in implantation, and interactions between NO and AEA could be of importance. We have previously observed that AEA increases uterine and deciduas NO production through iNOS activation in a mouse model of fetal resorption [20], [21].

Based on the above evidence, the principal aim of the present work was to study in an *in vitro* system if AEA modulated NOS activity at implantation and if the blastocyst participates of this interaction. Pseudopregnancy and ovariectomy-induced delayed implantation models were used as tools to understand the relative roles played by the embryo. The fact that AEA differently regulated NOS activity through CB1 and CB2 receptors and that this modulation depended on the presence of the blastocyst during implantation, contributes to better understand the significance of ligand – receptor signalling with AEA and NO as possible effectors in coordinating the series of events that leads to a successful pregnancy.

## Materials and Methods

### Ethics Statement

The experimental procedures reported here were approved by the Animal Care Committee of the Centro de Estudios Farmacológicos y Botánicos (CEFYBO - CONICET) and by the Institutional Committee for the Care and Use of Laboratory Animals, Permit Number: 2550/2010 (CICUAL, Comité Institucional para el Cuidado y Uso de Animales de Laboratorio) from the Facultad de Medicina (Universidad de Buenos Aires), and were carried out in accordance with the Guide for Care and Use of Laboratory Animals (NIH). All animals were provided by the animal facility of the Facultad de Odontología (Universidad de Buenos Aires).

### Animals

Female rats of the Wistar strain were housed in group cages under controlled conditions of light (12 h light, 12 h dark) and temperature (23–25°C). Animals received food and water *ad libitum*. Where mentioned, animals were sacrificed in a carbon dioxide chamber and all efforts were made to minimize suffering.

Virgin female rats were mated with fertile males of the same strain. The morning the spermatozoa were observed in the vaginal fluid was defined as day 1 of pregnancy. Under the conditions of our animal facilities, spontaneous term labor occurs on day 22 of gestation. Blastocysts enter the uterus lumen between days 4 and 5 of gestation and implantation begins during the afternoon of day 5. Rats on days 4 to 6 of pregnancy (peri-implantation) were sacrificed at 9:00–10:00 in the morning. On day 6 of pregnancy distinct macroscopically visible uterine swellings indicated the implantation sites. Uterine horns of rats on days 4–6 of pregnancy were excised and uterine horns on day 6 were separated into implantation and inter-implantation sites.

Different methods of manipulating pregnancy-related uterine changes (pseudopregnancy and ovariectomy-induced delayed implantation) were used as tools to understand the relative roles played by the embryo and ovarian hormones in modulating the changes in NOS activity in the pregnant rat. These models were previously established [16].

Briefly, for delayed implantation, rats were ovariectomized at 9:00–10:00 on day 4 of pregnancy. To maintain the implantation delay, rats were injected with progesterone (8 mg kg^−1^, s.c.) on days 5 and 6 of pregnancy. On day 7 of gestation, females were divided randomly into two groups and treated respectively with progesterone (8 mg kg^−1^, s.c.) or 17β-estradiol (0.4 mg kg^−1^, s.c.). Steroids were dissolved in 0.2 mL of corn oil. Rats were sacrificed at 9:00–10:00 on day 8 and their uteri were collected and stored at −70°C until used.

Pseudopregnancy (psp) could be induced in female rats treated with equine chorionic gonadotrophin (PMSG). In this model, the uterus undergoes all the normal changes that prepare it for implantation, but no embryos are present in the uterine lumen. Prepuber rats (25–28 days of age) received 50 iu PMSG i.p. [16], [22]. Day 1 of pseudopregnancy was considered 24 h after the injection. Females were sacrificed on days 4, 5 and 6 of psp. PMSG was disolved in saline.

### Total NOS enzyme assay

NOS enzyme activity was quantified by the modified method of Bredt & Snyder [23] which measures the conversion of [^14^C]-L-arginine into [^14^C]-L-citrulline. NO and L-citrulline are produced in equimolar amounts.

Uterine slices were weighted and where mentioned, they were incubated with different drugs (AEA, SR141716A, SR144528 or URB-597) in Krebs Ringer Bicarbonate solution (145 mM Na^+^, 6 mM K^2+^, 2 mM Ca^2+^, 1.3 mM^1^ Mg^2+^, 126.1 mM Cl^−^, 25.3 mM HCO_3_^−^, 1.3 mM SO_4_^2−^, 1.2 mM PO_4_^2−^) with 11 mM glucose. Then, tissues were homogenized (Ultra Turrax, T25 basic, IKA Labortechnik) and incubated at 37°C in an HEPES buffer (20 mM HEPES, 25 mM L-valine, 0.45 mM CaCl_2_, 100 mM DTT) containing 0.6 µCi ml^−1^ [^14^C]-L-arginine and 0.5 mM NADPH. After 15 min of incubation, samples were centrifuged for 15 min at 12000 g. They were then applied to a 1 mL DOWEX AG500-X column (Na^+^-form) and [^14^C]-L-citrulline was eluted in 2.5 mL of distilled water. The [^14^C]-L-citrulline radioactivity was measured by liquid scintillation counting. Protein concentration was determined by the Bradford method [24]. Enzyme activity was expressed as pmol L-citrulline mg protein^−1^ h^−1^.

### Polymerase chain reaction analysis

Uterine total RNA from rats on day 5 of pseudopregnancy was isolated using Trizol reagent according to the manufacturer's recommendations. RNA was thawed on ice, quantified spectrophometrically at 260 and 280 nm and RNA quality assessed using ethidium bromide-stained gels. RNA with a 260∶280 ratio of 1.8 and above was further treated with RNAse free DNAse I to digest contaminating genomic DNA. First strand cDNA was synthesized from total RNA (3 µg) using Moloney murine leukemia virus reverse transcriptase (MMLV-RT) and random primers according to the manufacturer's recommendations (Invitrogen, Buenos Aires, Argentina) in the presence of ribonuclease inhibitor. After first strand synthesis, polymerase chain reaction (PCR) was performed with specific intron spanning primers. PCR primers are specified in Table 1. The PCR conditions in all cases started with a denaturation step at 94°C for 5 min and followed by up to 30 cycles of denaturation, annealing and primer extension (Table 1). PCR products were resolved in 2% agarose gels and visualized by ethidium bromide staining. Photographs were taken using a digital camera Olympus C-5060 and analyzed using the Image J (open source) software package. Data were expressed as the relative amount of CB1 or CB2 versus β-actin mRNA.

**Table 1:** Primer sequences and PCR conditions.

mRNA target	Primers	Conditions
β-actin	Sense 5′-GCCATGTACGTAGCCATCC-3′	94°C, 5 min
	Antisense 5′-CTCTCAGCTGTGGTGGTGAA-3′	59°C, 30 seg
		72°C, 1 min
Cb1	Sense 5′-CGTAAAGACAGCCCCAATGT-3′	94°C, 5 min
	Antisense 5′-TACCTGTCGATGGCTGTGAG-3′	59°C, 30 seg
		72°C, 1 min
Cb2	Sense 5′-CCTGTTGAAGATCGGCAGCG-3′	94°C, 5 min
	Antisense 5′-GGTAGGAGATCAACGCCGAG-3′	57°C, 30 seg
		72°C, 1 min

Cb1: cannabinoid receptor type-1; Cb2: cannabinoid receptor type-2.

### Real Time RT-PCR analysis

RNA messenger expression in the uterus from rats pregnant on days 4, 5 and 6 of gestation was analyzed by real time RT-PCR. Real time RT-PCR was performed on RG6000 (Corvette) using Master Mix, gene specific primers (Table 1) and 5 µl of cDNA as template. The PCR conditions in all cases started with a denaturation step at 94°C for 5 min and followed by up to 40 cycles of denaturation, annealing and primer extension (Table 1). CB1 and CB2 mRNA levels were corrected to the levels of rat β-actin using the 2-^ΔΔCt^ method [25] and normalized to the ratio produced in the samples from the day 4 of pregnancy.

### CB1 and CB2 identification by western-blotting

Cannabinoid receptors proteins from the uterus of pregnant and psp rats were determined by western blot analysis using a CB1 or a CB2 specific antibody. Uterine slices frozen at −70°C were incubated in triple-detergent buffer (PBS pH = 7.4 with 0.02% w/v sodium azide, 0.1% w/v SDS, 1% v/v Nonidet P-40, 0.5% v/v sodium deoxycholate) containing 10 µg ml^−1^ leupeptin, 2 µg ml^−1^ aprotinin, 100 µg ml^−1^ soybean-trypsin inhibitor, 1 mmol l^−1^ EDTA, 1 mg ml^−1^ benzamidine, 10 µg ml^−1^ DTT and 1 mg ml^−1^ caproid acid. Tissues were homogenized (Ultra Turrax, T25 basic, IKA Labortechnik), sonicated for 30 s (Ultrasonic Cell Disrupter, Microson, Heat systems Inc.) and centrifuged for 30 min at 12000 g. Protein determination was assayed by the Bradford method [24] using bovine serum albumin as standard. Equal amount of proteins (100 µg/lane) were separated in 10% (CB1) or 12% (CB2) w/v SDS-PAGE and subsequently transferred to nitrocellulose membranes. Non-specific binding sites of the membranes were blocked using 5% w/v dried non-fat milk in PBS pH = 7.4. Non-specifically bound antibody was removed by washing three times with PBS containing 0.1% v/v Tween-20. Membranes were incubated with primary CB1 (1∶250 in PBS) or CB2 (1∶200 in PBS) antibodies followed by a goat anti-rabbit horseradish peroxidase-conjugated IgG (1∶8000 for CB1 and 1∶5000 for CB2 in PBS containing 5% w/v dried non-fat milk). Immunoreactive bands were visualized and photographed using Image Quant Software (GE Healthcare, Buenos Aires, Argentina). Brain and spleen homogenates were used as positive controls for CB1 and CB2 respectively. Immunoreactive specificity was assessed by omitting the first antibodies. Protein bands were identified by molecular weight markers. β-actin was used as loading control. The intensity of bands was determined using the Image J software package (open source). Results were expressed as relative optic density CB1 or CB2 receptor/β-actin.

### Light microscopy and immunohistochemistry

Uteri from pregnant rats on days 4, 5 and 6 of gestation were removed and fixed overnight in paraphormaldehyde 4% in phosphate-buffered saline (PBS) 0.1 M (pH 7.4). The tissue sections were dehydrated and embedded in paraffin. The paraffin block was orientated to enable the implantation sites to be sectioned transversally. Sections of 5 µm were made by a microtome (Leica RM 2125, Wetzlar, Germany) and mounted on 2% silane-coated slides. The sections were stained with hematoxylin–eosin, and observed by light microscopy (Nikon Eclipse 200, NY, USA) to determine general tissue morphology and to identify the different cell types present. For immunohistochemistry studies, the samples were blocked of endogenous peroxidase with hydrogen peroxide 0.3% v/v in methanol for 10 min and rinsed with PBS. The slides were pre-incubated with non-immune rabbit serum diluted in PBS (1∶100) in a humidity chamber at room temperature for 1 h. Then, the slices were incubated at 4°C overnight in a humidity chamber with a polyclonal rabbit antibody directed against CB1 or CB2 (1∶50 in PBS). The immunoperoxidase technique was then performed following the protocol for the RTU Vectastain kit (Vector, Peterborough, UK). The antigen was revealed by diaminobenzidine (DAB, Vector, Peterborough, UK). Finally, the sections were dehydrated, counterstained and mounted for observation. In each experimental immunohistochemical run, sections from all days of pregnancy (days 4 to 6) were included.

### Drugs and chemicals

[^14^C]-L-arginine monohydrocloride (specific activity 317 mCi mmol^−1^) was provided by Amersham Corporation (Arlington Heights, IL, USA). Optiphase-3 scintillation solution was from Perkin Elmer (ETC, Buenos Aires, Argentina). Luminol (Fluka), p-coumaric acid (Fluka), anandamide, 17β-estradiol, progesterone, NADPH, HEPES, valine, Dowex AG500-X column (Na^+^-form) and western blot detergents and inhibitors were purchased to Sigma Chemical Company (Buenos Aires, Argentina) and Bio Rad (Tecnolab, Buenos Aires, Argentina). Chemiluminescence detection solutions were from Romek Laboratories (Buenos Aires, Argentina). Trizol reagent was from Genbiotech (Buenos Aires, Argentina). RNAse free DNAse I, Moloney Murine Leukemia virus reverse transcriptase (MMLV-RT) and random primers were purchased from Invitrogen (Buenos Aires, Argentina). Master Mix for real time RT-PCR was from Promega (Biodynamics, Buenos Aires, Argentina). Polyclonal rabbit antibody against CB1 was from Biomol (Enzo Life Sciences, Miami, USA). CB2 antibody was purchased from Abcam (Migliore Laclaustra, Buenos Aires, Argentina). Goat anti-rabbit horseradish peroxidase-conjugated IgG was from Jackson ImmunoResearch Laboratories, Inc. (SERO-IMMUNO DIAGNOSTICS, INC, Tucker, GA, USA). PMSG (equine chorionic gonadotrophin, NOVORMON®) was kindly provided by Syntex S.A. (Buenos Aires, Argentina). CB1 selective antagonist (SR141716A, N-piperidino-5-(4-chlorophenyl)-1-(2,4-dichlorophenyl)-4-methyl-3-pyrazole carboxamide) [26] and CB2 selective antagonist (SR144528, N-[1(S)-endo-1,3,3-trimethyl-bicyclo[2.2.1]heptan-2-yl]-5-(4-chloro-3-methylphenyl)-1-(4-methyl-benzyl)-pyrazole-3-carboxamide) [27] were kind gifts from Sanofi-Aventis Recherche (Montpellier, France). URB-597 was from Cayman Chemical Company (Migliore Laclaustra, Buenos Aires, Argentina). All other chemicals were analytical grade.

### Statistical analysis

Statistical analysis was performed using the GraphPad Prism Software (San Diego, CA, USA). Comparisons between values of different groups were performed using one way ANOVA (analyze of variance). Significance was determined using Tukey's multiple comparison tests for unequal replicates. A number of 4–6 animals were used for each treatment. All values presented in this study represent means ± S.E.M. Differences between means were considered significant when p was 0.05 or less.

## Results

### NOS activity was modulated during peri-implantation

**Figure 1:**
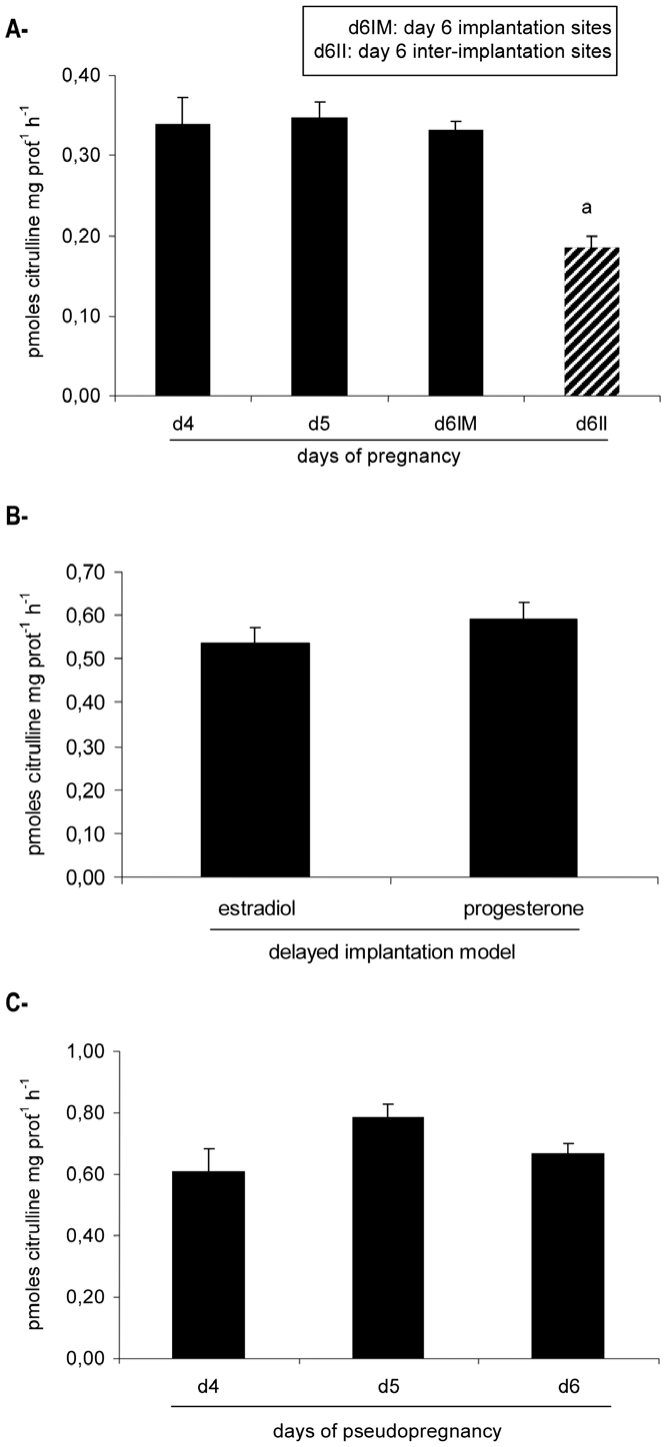
NOS activity in the rat uterus during peri-implantation. NOS activity was determined (A) on days 4 (d4), 5 (d5) and 6 of gestation (implantation (d6IM) and inter-implantation sites (d6II) were assessed separately on day 6 of pregnancy), (B) in the delayed implantation model and (C) during pseudopregnancy (psp). NOS activity is expressed as pmoles citrulline mg prot^−1^ h^−1^. a: p<0,001 vs the rest. N = 4–6 for each point. d4: day 4, d5: day 5, d6: day 6, IM: implantation sites, II: inter-implantation sites.

Uterine tissue from pregnant rats presented detectable NOS activity in all the days analyzed (Figure 1A). NOS activity remained constant on days 4 and 5 of gestation and at implantation sites from day 6 of gestation. However, a significant decrease happened at inter-implantation sites from day 6. Thus, NOS activity was higher in the receptive pre-implantation uterus and at the sites of embryo implantation compared to the inter-implantation sites.

As NOS activity was differentially regulated between implantation and inter-implantation sites, we decided to investigate if this modulation was supported by a crosstalk between the blastocyst and the uterus and/or by ovarian hormones. Thus, we studied NOS activity in the delayed implantation model and during pseudopregnancy (see methods). In delayed implantation rats, there was no difference between the treatments with 17β-estradiol, that activated implantation, or progesterone, that maintained the embryo and the uterus quiescent (Figure 1B). In pseudopregnancy, the uterus underwent the same changes observed in control pregnancies, but embryos were absent. Results show that in the absence of a blastocyst, NOS activity remained constant during psp and there was no drop as observed in normal gestation (Figure 1C).

### CB1 and CB2 mediated AEA differential effects on NOS activity

Previous results from Guo et al. [14], Paria et al. [15] and our group [16] demonstrate that on day 6 of pregnancy, implantation sites show lower AEA synthesis compared to inter-implantation sites. Since AEA synthesis presents an inverted pattern compared to NOS activity described above, we hypothesized that low AEA levels near to and at implantation sites might increase NOS activity, while high AEA levels at inter-implantation sites might decrease it. We decided to investigate this issue and to determine the relative role of the blastocyst in the interaction AEA-NO.

**Figure 2:**
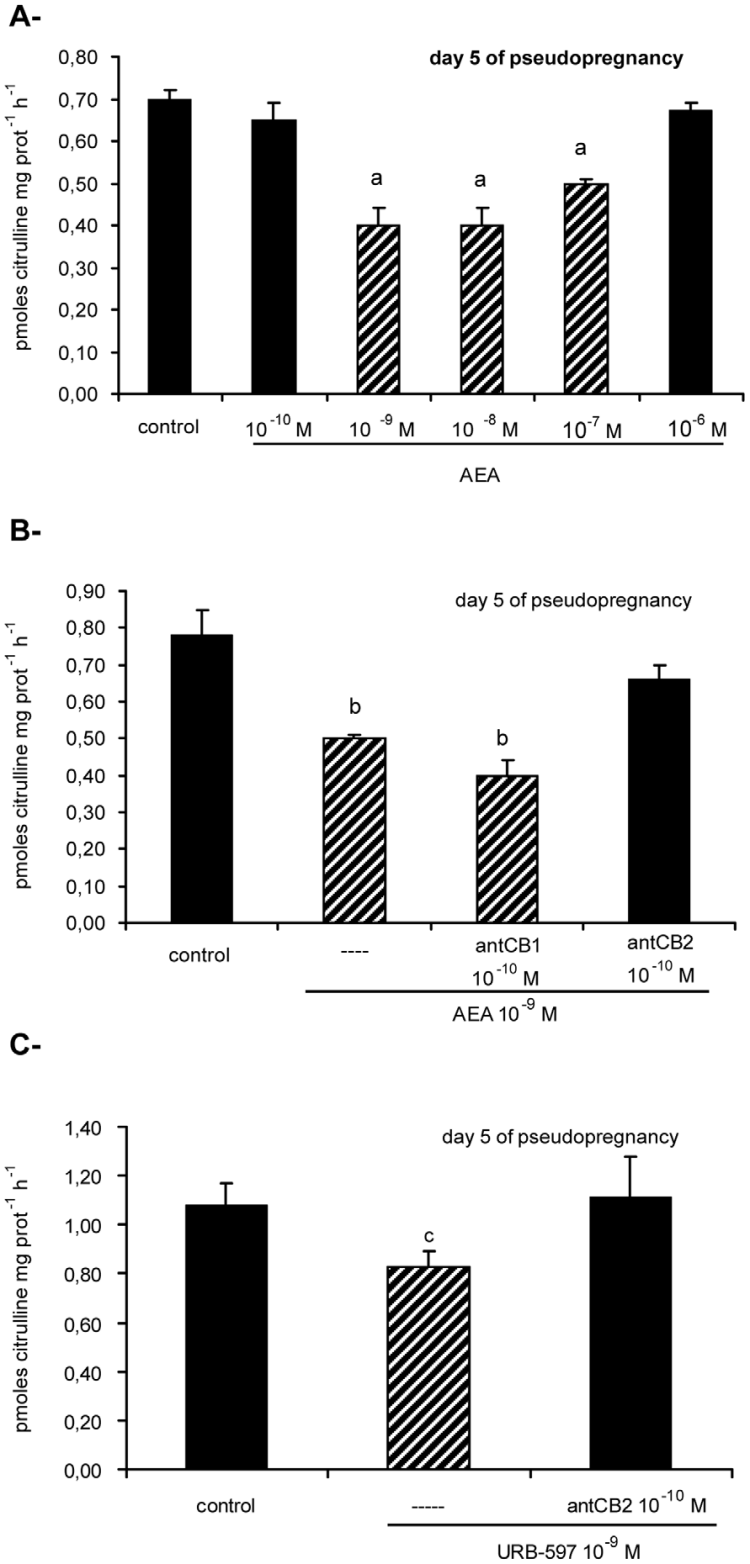
Effect of AEA on NOS activity on day 5 of pseudopregnancy. (A) Concentration-response curve of anandamide (AEA). (B) Effect of selective cannabinoid receptors antagonists on AEA inhibitory action. (C) Effect of URB-597 (a selective inhibitor of FAAH, the enzyme that degrades AEA) and selective cannabinoid receptors antagonists. NOS activity is expressed as pmoles citrulline mg prot^−1^ h^−1^. a: p<0,001 vs the rest, b: p<0.001 vs the rest, c: p<0.05 vs the rest. N = 4–6 for each point. antCB1: CB1 selective antagonist (SR141716A), antCB2: CB2 selective antagonist (SR144528).

In order to determine optimal incubation conditions, a concentration-response curve was carried out using day 5 psp uterine tissue. Incubation during 15 min with AEA 10^−9^ M, 10^−8^ M and 10^−7^ M inhibited NOS activity (Figure 2). AEA 10^−6^ M and 10^−10^ M did not modify NOS activity. The incubation time was selected due to AEA short half life [28]. In subsequent experiments we incubated the tissues with AEA 10^−9^ M, which is the physiological concentration reported in different reproductive fluids [12], [14], [29] and the lowest concentration that showed effect.

To study which receptors were mediating AEA effect in the pseudopregnant model, uterine strips from day 5 of psp were pre-incubated for 30 min with SR141716A 10^−10^ M (a CB1 selective antagonist) or SR144528 10^−10^ M (a CB2 selective antagonist). Afterwards tissues were incubated during 15 min with AEA 10^−9^ M in the presence of the antagonists. Based on both binding and functional data, SR141716A and SR144528 at the selected concentration are highly potent and selective antagonists for the CB1 and CB2 receptors respectively [30], [31]. Nor SR141716A 10^−10^ M neither SR144528 10^−10^ M incubated alone presented any effect on NOS activity (Table 2). While SR141716A 10^−10^ M, the CB1 selective antagonist, did not modify AEA action, SR144528 10^−10^ M, the CB2 selective antagonist, reverted AEA inhibitory effect on NOS activity in the pseudopregnant model (Figure 2B). To determine the effect of endogenous AEA, uterine strips from day 5 of psp were incubated for 30 min with URB-597, a FAAH selective inhibitor. As FAAH is the enzyme that degrades AEA, the treatment with URB-597 increases AEA endogenous concentration. We observed that URB-597 10^−9^ M inhibited NOS activity (Figure 2C). As previously observed for exogenous AEA, pre-incubation for 30 min with SR144528 10^−10^ M, the selective CB2 antagonist, reversed URB-597 inhibitory effect on NOS activity. Thus, in the absence of the blastocyst, AEA inhibited NOS activity through CB2 receptors.

**Table 2:** Effect of cannabinoid receptors selective antagonists on NOS activity.

	NOS activity
control	0.90±0.10
SR141716A 10^−10^ M	0.90±0.10
SR141716A 10^−9^ M	0.75±0.10
SR144528 10^−10^ M	0.90±0.20
SR144528 10^−9^ M	0.80±0.20

Cannabinoid receptors selective antagonists SR141716A (type 1, CB1) or SR144528 (type 2, CB2) were incubated alone for 30 min with day 5 pseudopregnant rat uterus and NOS activity was determined. Results are expressed as pmoles citrulline mg prot^−1^ h^−1^. N = 4–6 for each point.

**Figure 3:**
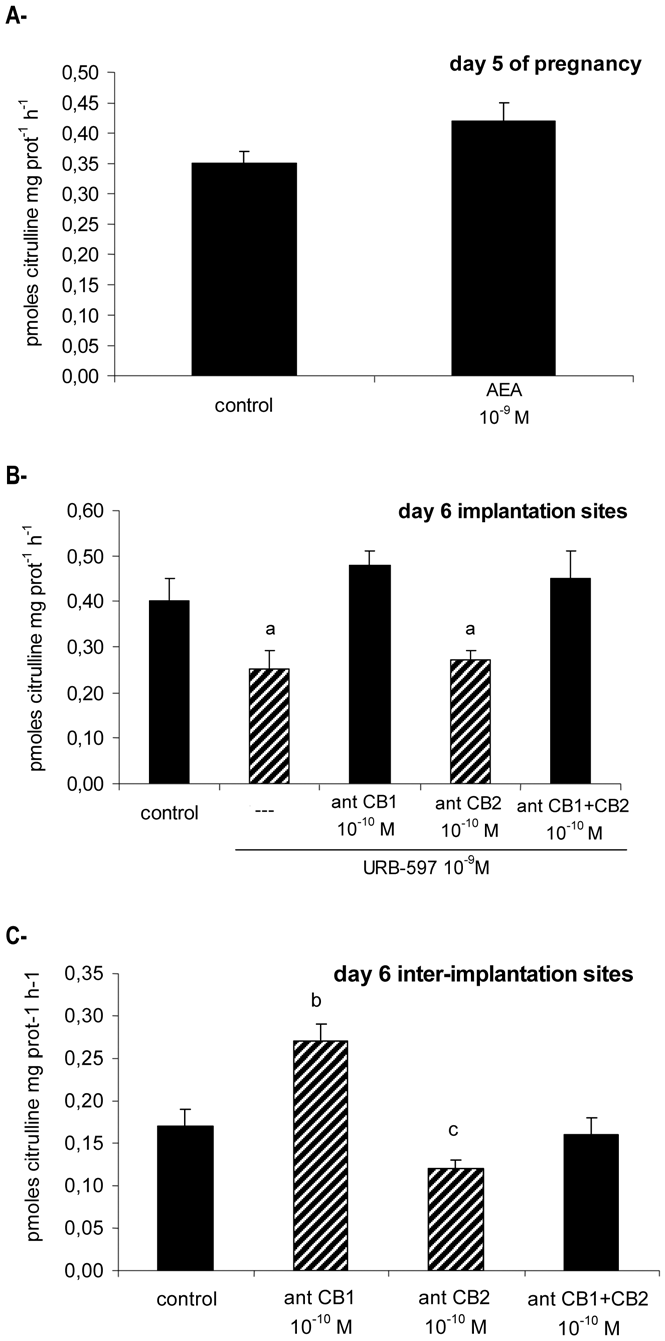
Effect of AEA on NOS activity in the uterus from pregnant rats. (A) Uterine tissue from day 5 pregnant rats was incubated for 30 min with AEA 10^−9^ M. (B) Implantation sites from day 6 of pregnancy were incubated for 15 min with AEA 10^−9^ M alone or pre-incubated with selective cannabinoid receptors antagonists. (C) Inter-implantation sites from day 6 of pregnancy were incubated with selective cannabinoid receptors antagonists. NOS activity is expressed as pmoles citrulline mg prot^−1^ h^−1^. a: p<0,001 vs the rest, b: p<0,001 vs control, antCB2 and antCB1+CB2, c: p<0.05 vs control and antCB1+antCB2. N = 4–6 for each point. antCB1: CB1 selective antagonist (SR141716A), antCB2: CB2 selective antagonist (SR144528).

When the uterus obtained from rats pregnant on day 5 were incubated with AEA 10^−9^ M, it did not exert any effect on NOS activity (Figure 3A). In contrast to what was observed on day 5 of psp, AEA was not able to regulate NOS activity just before the blastocyst begins the process of implantation (day 5 of pregnancy).

Other authors [14], [15] and our group [16] have previously observed that mouse and rat implantation sites show low AEA synthesis, while it is increased at the inter-implantation sites. Thus, implantation sites from day 6 pregnant rats were incubated for 30 min with URB-597 10^−9^ M to increase endogenous AEA levels. We observed that URB-597 diminished NOS activity and that this effect was completely reverted when the implantation sites were pre-incubated for 30 min with the selective CB1 antagonist SR141716A 10^−10^ M (Figure 3B). In order to study endogenous AEA effect on day 6 inter-implantation sites, this tissue was incubated for 30 min with SR141716A 10^−10^ M or SR144528 10^−10^ M. We observed that while CB1 selective antagonist (SR141716A 10^−10^ M) increased NOS activity, the CB2 selective antagonist (SR144528 10^−10^ M) decreased it (Figure 3C). The incubation with both CB1 and CB2 antagonists did not modify NOS activity with respect to the control. These results showed that once implantation begins, at the implantation sites, AEA inhibited NOS activity through CB1 receptors. However, at the inter-implantation sites, AEA presented a dual effect: it inhibited NOS activity through CB1, while it increased NOS via CB2 receptors.

### Cannabinoid receptors expression and localization in the rat uterus

Finally, we decided to investigate CB1 and CB2 expression and localization in order to better understand the relative roles played by these receptors in the effect of AEA on NOS activity. Also to our knowledge, there are still no reports about the expression of CB2 protein in the rat uterus during the peri-implantation period, and here we found that CB2 mediated some of the actions of AEA on NOS activity.

**Figure 4:**
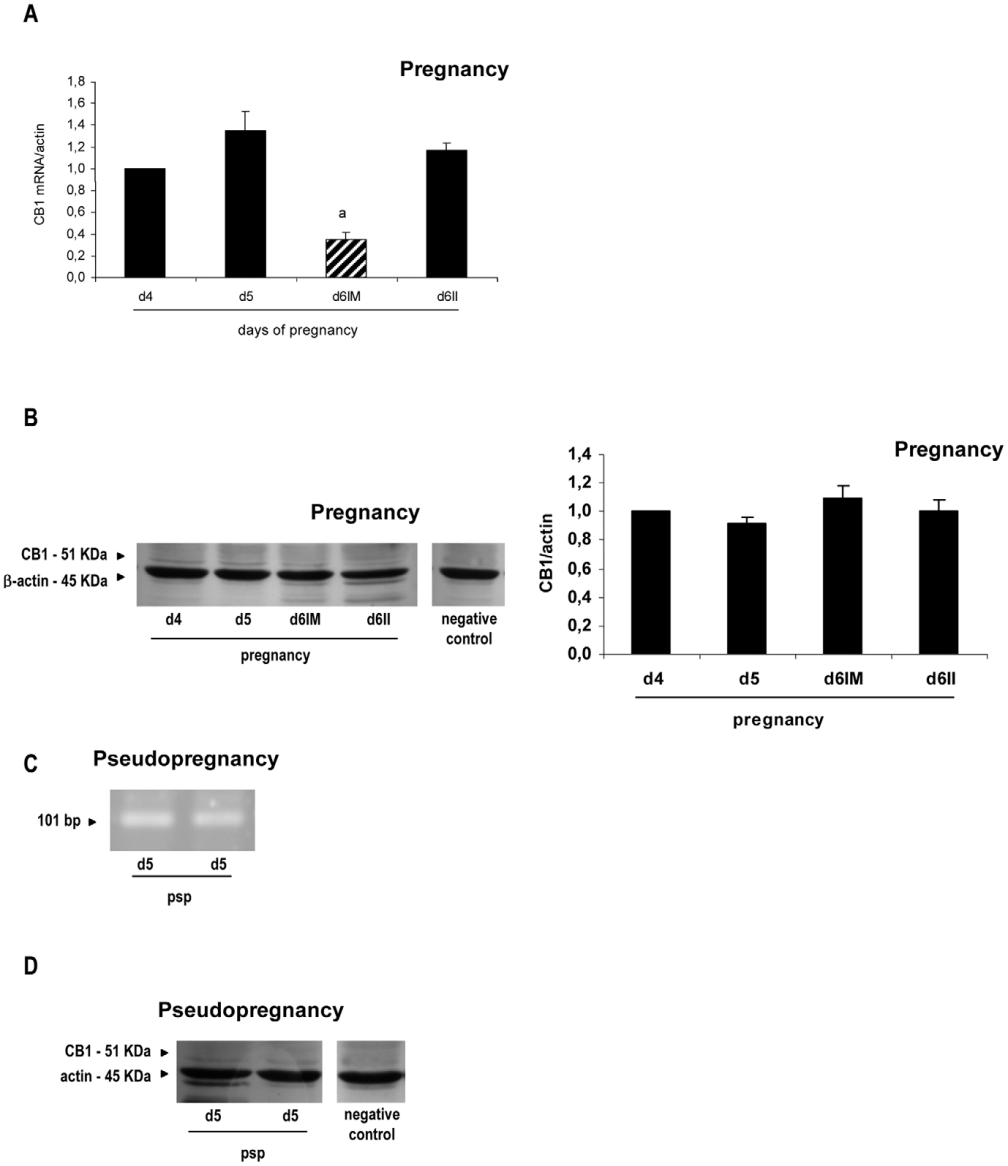
Expression of cannabinoid receptor type-1 at implantation and pseudopregnancy. Cannabinoid receptor type-1 (CB1) messenger (A and C) and protein (B and D) were detected during peri-implantation (A and B) and on day 5 of pseudopregnancy (C and D). Results are shown as means ± S.E.M. N = 4–6 for each point. a: p<0.001 vs the rest. d4: day 4, d5: day 5, d6: day 6, IM: implantation sites, II: inter-implantation sites, psp: pseudopregnancy.

CB1 messenger was amplified by real time RT-PCR in control pregnancy and identified by RT-PCR in pseudopregnancy as a single band at 101 bp. CB1 messenger was clearly regulated showing a significant decrease at the implantation sites from day 6 of gestation (p<0.01) compared with days 4 and 5 of pregnancy and with the inter-implantation sites from day 6 (Figure 4A). CB1 protein was detectable as a single band at the expected molecular mass of 51 KDa during pregnancy [32]. However, it seemed not to be regulated in the rat uterus as was CB1 mRNA (Figure 4B). On day 5 of pseudopregnancy, both CB1 messenger and protein were detected (Figure 4C and 4D).

**Figure 5:**
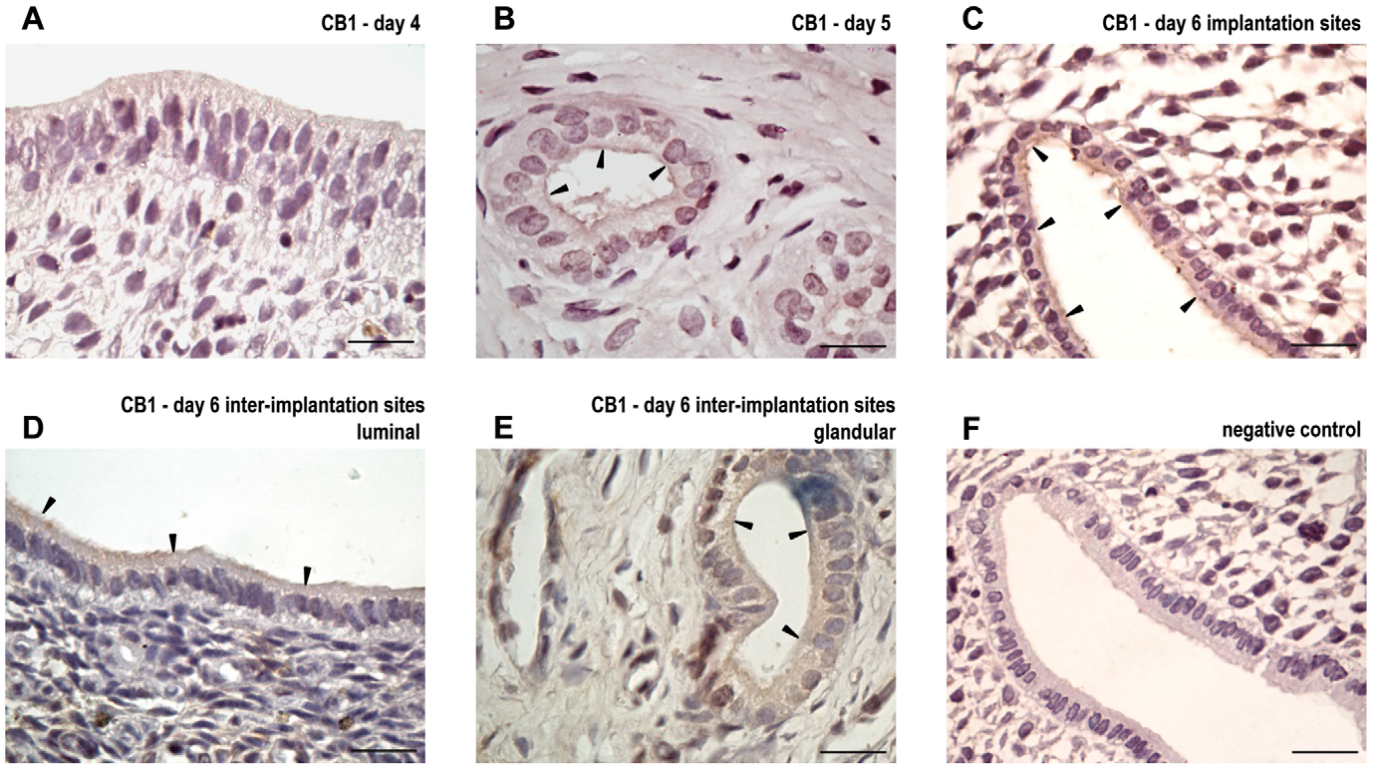
Localization of cannabinoid receptor type 1 during implantation. Immunolocalization of cannabinoid receptor type-1 (CB1) in uteri from day 4 (A), day 5 (B), day 6 implantation sites (C) and day 6 inter-implantation sites (D: luminal, E: glandular). Tissue sections were processed by the immunoperoxidase technique using a polyclonal antibody directed against CB1. No staining was observed in the luminal and glandular epithelium when the first antibody was omitted (F). Black arrows denote specific staining. The scale bar indicates 20 µm.

With respect to CB1 localization in pregnancy, it was detectable in the endometrium obtained from rats on days 5 and 6 of gestation, but we were not able to detect it on day 4 of pregnancy (Figure 5A). On day 5, CB1 was immunoreactive in the apical membrane of the glandular epithelium (Figure 5B). While in the implantation sites CB1 was localized to the apical membrane and subapical cytoplasm of the luminal epithelium (Figure 5C), in the inter-implantation sites it was also detected in the glandular epithelium of the endometrium (Figures 5D and 5E).

**Figure 6:**
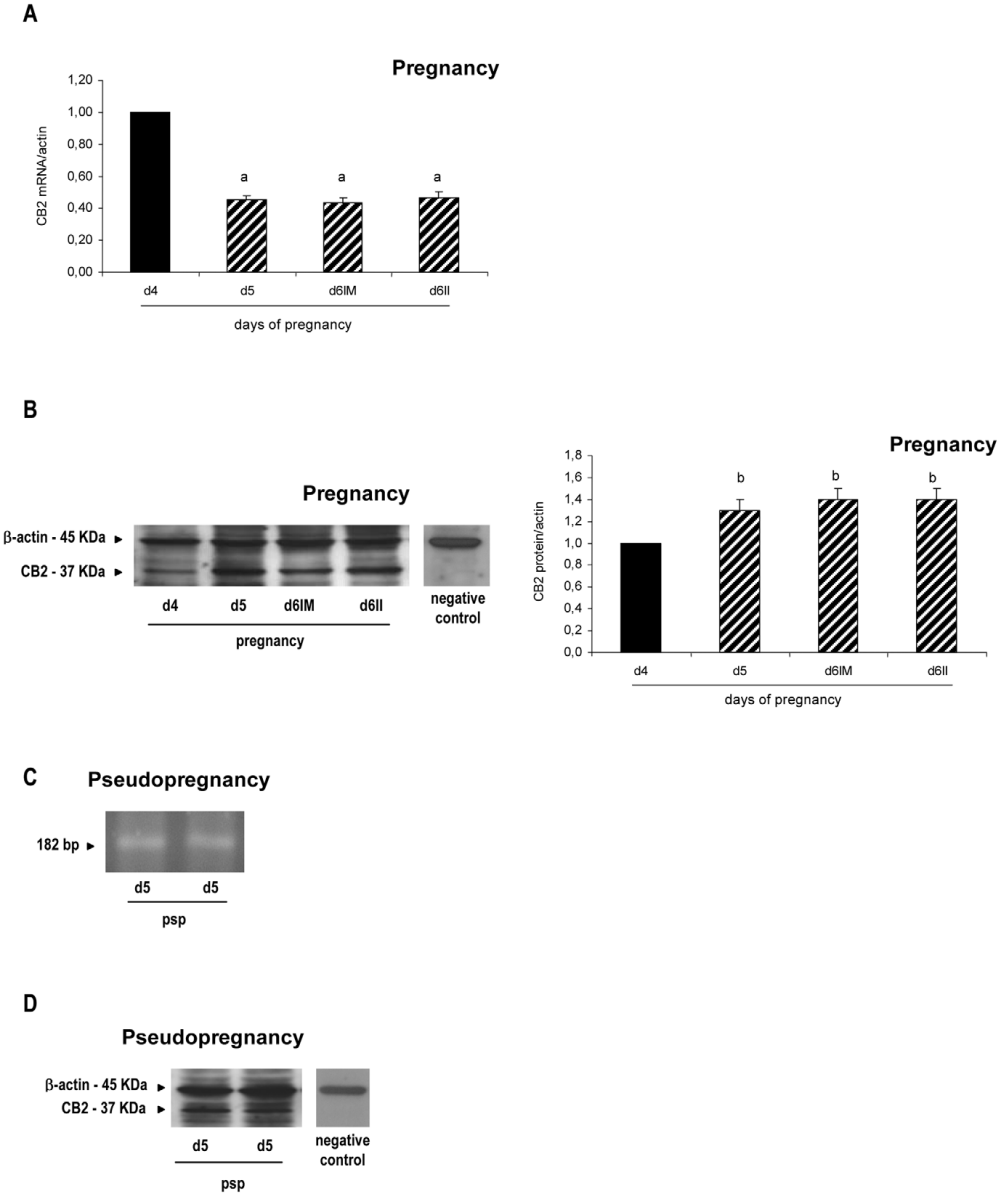
Expression of cannabinoid receptor type-2 at implantation and pseudopregnancy. Cannabinoid receptor type-2 (CB2) messenger (A and C) and protein (B and D) were detected during peri-implantation (A and B) and on day 5 of pseudopregnancy (C and D). Results are shown as means ± S.E.M. N = 4–6 for each point. a: p<0.01 vs day 5, b: p<0.05 vs day 4. d4: day 4, d5: day 5, d6: day 6, IM: implantation sites, II: inter-implantation sites, psp: pseudopregnancy.

CB2 messenger was also amplified by real time RT-PCR at pregnancy and identified by RT-PCR in psp as a single band at 182 bp. During peri-implantation, we observed that CB2 messenger decreased on days 5 and 6 compared to day 4 (p<0.001) and that there was no difference between the implantation and the inter-implantation sites (Figure 6A). CB2 protein expression was readily detectable at pregnancy and it appeared as a single band at approximately 37 KDa (Figure 6B). One immunoreactive band of higher molecular weight was also observed that most likely corresponded to a glycosylated form of the receptor [32]. CB2 protein expression increased on days 5 and 6 of gestation when compared with day 4 of pregnancy (p<0.05, Figure 6B). On day 6, after the blastocyst is attached, there were no spatial differences between the implantation and the inter-implantation sites. On day 5 of pseudopregnancy, CB2 messenger and protein were readily detected (Figures 6C and 6D).

**Figure 7:**
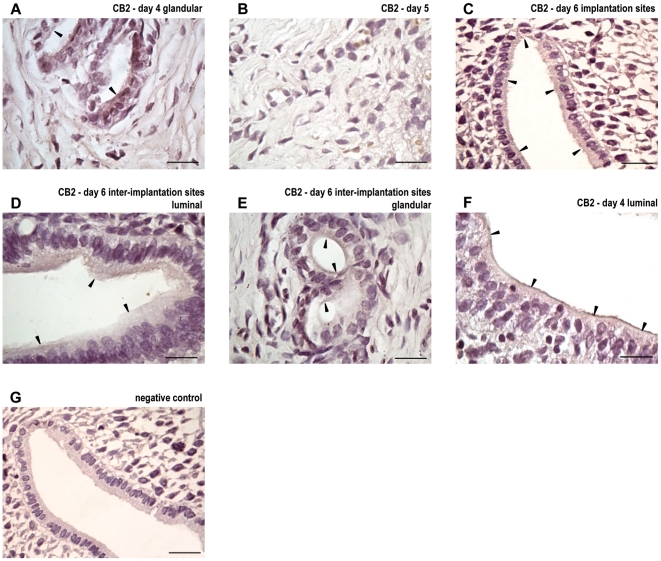
Localization of cannabinoid receptor type 2 during implantation. Immunolocalization of cannabinoid receptor and type-2 (CB2) in uteri from day 4 (A: luminal and B: glandular), day 5 (C), day 6 implantation sites (D) and day 6 inter-implantation sites (E: luminal, F: glandular). Tissue sections were processed by the immunoperoxidase technique using a polyclonal antibody directed against CB2. No staining was observed in the luminal and glandular epithelium when the first antibody was omitted (G). Black arrows denote specific staining. The scale bar indicates 20 µm.

Finally, we decided to investigate the localization of CB2 during pregnancy. Although CB2 protein was readily detectable in the endometrium on days 4 and 6 of gestation, we were not able to observe CB2 expression on day 5 of gestation (Figure 7C). On day 4, CB2 was localized to the luminal (Figure 7A) and glandular (Figure 7B) epithelium both in the apical membrane and in the subapical cytoplasm. In the implantation sites, CB2 was observed only in the luminal epithelium at the apical membrane and subapical cytoplasm (Figure 7D). In the inter-implantation sites from day 6 of gestation, CB2 was localized to the apical membrane and subapical cytoplasm in the luminal epithelium (Figure 6E) and to the apical membrane in the glandular epithelium (Figure 6F). No staining was observed in the luminal or glandular epithelium when the first antibodies were omitted (Figures 6G).

## Discussion

In the present work we observe that in the rat uterus NOS activity is increased during pre-implantation and at implantation sites compared to inter-implantation sites. Interestingly, AEA modulates NOS activity through cannabinoid receptors and this regulation depends on the presence of the blastocyst. Also, we describe for the first time the expression and localization of CB1 and CB2 receptors during implantation in the rat uterus.

Regulation of NOS activity described here coincides with previously reported iNOS and eNOS expression in early gestation [4], [33], [34]. The fact that NOS activity is higher at implantation sites suggests that NO has a role specifically where blastocysts attach to the endometrium. Purcell and colleagues described that eNOS and iNOS proteins are localized in the endometrial decidua and stromal cells as well as in the myometrium and blood vessels that irrigate the uterine tissue [4]. Neuronal NOS is restricted to the myometrium. Thus, it is possible that blastocysts might be increasing NO production by regulating eNOS and iNOS, the isoforms that are expressed in the cellular types in direct contact with the embryo.

Two processes are fundamental during the establishment of pregnancy: the increase in vascular permeability and decidualization. NO is a potent vasodilator and a well known mediator of vascular permeability [35]. Besides, NO augments the expression of some specific matrix metalloproteinases [36] which are known to participate in tissue remodeling. Thus, our results reinforce the notion that increased NOS activity in the receptive uterus and specifically at implantation sites might be contributing to neovascularization and tissue remodeling during trophoblast invasion.

Novaro and colleagues described that NOS activity increases in pre-implantation days and then declines on day 6 of gestation [5]. Differences with our results could be due to the way in which results were expressed and because we differentiated implantation from inter-implantation sites.

In this study, we use ovariectomy – induced delayed implantation and pseudopregnant models as tools to understand the relative roles played by the embryo and ovarian steroid hormones in modulating NOS activity in the rat pregnant uterus. The correlation between NOS and receptive/non-receptive uterine phases emphasizes the notion that NOS could be locally regulated in the uterus by pre-implanting and implanting blastocysts, supporting the possibility that the embryo maintained high NOS activity both in the receptive uterus and at implantation. Moreover, the fact that 17β-estradiol and progesterone produce similar levels of NOS activity supports the notion that dormant and activated embryos are capable of modulating this enzyme. The differences in NOS activity observe between the three models used in the present work, probably corresponds to the hormonal treatments employed, which mimics the hormonal milieu that occurs during normal pregnancy but are not exactly the same. In accordance with our results, it has been previously informed that NOS activity is differently regulated in a rat model in which embryo access to the uterus is impaired [5]. All together our results suggest that the presence of the embryo within the uterine lumen and at implantation sites, together with ovarian hormones, modulate NOS activity during early gestation.

**Figure 8:**
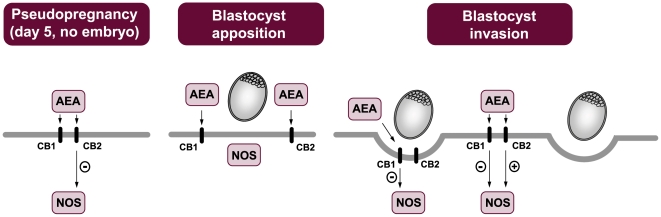
Summary of cannabinoid receptors mediated effects of AEA on NOS activity. For details see description in the Discussion section.

Mouse uterus contains by far the highest levels of AEA detected in any mammalian tissue [12], [14]. The changing levels of AEA with changing pregnancy status are consistent with a possible role for this lipid molecule in early pregnancy. In fact, AEA synthesis previously described [14]–[16] is negatively correlated with the activity of the NOS enzyme describe in the present study. We observe that the presence of the blastocyst and its state of activation participate in the regulation that AEA plays on NOS activity. In Figure 8 we describe that in the pseudopregnant model, when the blastocyst is absent, AEA inhibits NOS activity by binding to CB2 receptors. However, on day 5 of pregnancy, when the blastocyst apposites over the endometrial endothelium, AEA does not modify NOS activity. Once embryo invasion begins, AEA inhibits NOS via CB1 receptors at the sites of implantation. In the inter-implantation sites, AEA exerts a dual effect: it inhibits NOS through CB1 and stimulates NOS activity via CB2. Recently, we have reported that in the placenta obtained from pregnant rats, AEA also inhibits NOS activity and that this effect is reversed by CB2 antagonists [37]. An interesting review has been published, in which the author discussed the different signalling pathways triggered by CB1 and CB2 in reproduction, especially those controlling the intracellular tone of nitric oxide [38]. Furthermore, it has been described in different systems that while CB1 activates NOS, CB2 inhibits it [17], [18]. The opposite effect of CB1 versus CB2 on NO release might be relevant for the *in vivo* control of reproduction, since NO plays several roles in female fertility [39].

As there are no studies describing the expression pattern of cannabinoid receptors during the peri-implantation period in the rat and in order to gain insight into the role of these receptors in the transduction of crucial signals during implantation, we decided to describe the temporal pattern of expression and localization of the anandamide-binding receptors CB1 and CB2 in the rat uterus. Our results describe for the first time the presence and regulation of CB1 and CB2 mRNA and protein in the luminal and glandular endometrium of the rat uterus during the peri-implantation period.

CB1 messenger clearly decrease at the implantation sites from day 6 of pregnancy. Although there was no difference after the blastocyst enters the uterus, there is a clear modulation once implantation begins, suggesting that the blastocyst would be regulating CB1 mRNA. When we analyze the expression of CB2 messenger, we observe that once the blastocyst enters the uterus (day 5 of pregnancy), its level remains constant, and contrary to what happens with CB1 mRNA, there is no difference between the implantation and the inter-implantation sites.

CB1 protein is not modulated during gestation as is CB1 messenger. There is no difference after the embryo enters the uterus or between the implantation and the inter-implantation sites. It is possible that as real time RT-PCR technique is more sensible than western blot analyses, the decrease in CB1 expression at the implantation sites could not be detected at the protein level. With respect to CB2 protein, it presents a similar pattern as CB2 mRNA: after the embryo enters the uterus (day 5) the protein level of CB2 remains constant.

Using Northern blot hybridization and reverse transcription-PCR, the group of Das and colleagues were the first ones to demonstrate that CB1 mRNA, but not CB2 mRNA, is expressed in the mouse uterus [40]. Later, in 1998, Buckley described by in situ hybridization the presence of both CB1 and CB2 mRNA in the rat placental cone and uterine smooth muscle from days of gestation 8 to 12 [41]. Recently, it has been described that CB receptors (CBRs) messenger and protein are expressed in the rat decidualized cells and placenta during mid and late gestation [42], [43].

Overall, our results suggest that the blastocyst intrinsic program might operate in conjunction with ovarian hormones regulating NOS activity via cannabinoid receptors in a specific manner during implantation. Critical changes in CBRs in the maternal side of the fetal-maternal interface would occur during implantation and finally influence the outcome of gestation. We hypothesize that the degree of CBRs activation together with the group of molecules activated downstream would finally determine the function of CB1 and CB2 in each zone of the uterus. AEA levels close to and at implantation sites modulate NOS activity and thus NO nitric oxide production, fundamental for implantation, via CBRs, and this depends on the presence of the blastocyst, establishing CBRs as an interesting and novel target for the treatment of implantation deficiencies. On a final note, it should be recalled that blastocysts' triggering specific signalling pathways may exist, which could impact reproductive events in yet-unknown ways.
